# Real-Time Forest Fire Detection by Ensemble Lightweight YOLOX-L and Defogging Method

**DOI:** 10.3390/s23041894

**Published:** 2023-02-08

**Authors:** Jiarun Huang, Zhili He, Yuwei Guan, Hongguo Zhang

**Affiliations:** 1 School of Resources and Environment, University of Electronic Science and Technology of China, Chengdu 611731, China; 2 Glasgow College, University of Electronic Science and Technology of China, Chengdu 611731, China

**Keywords:** forest fire detection, convolutional neural network, lightweight, dark channel

## Abstract

Forest fires can destroy forest and inflict great damage to the ecosystem. Fortunately, forest fire detection with video has achieved remarkable results in enabling timely and accurate fire warnings. However, the traditional forest fire detection method relies heavily on artificially designed features; CNN-based methods require a large number of parameters. In addition, forest fire detection is easily disturbed by fog. To solve these issues, a lightweight YOLOX-L and defogging algorithm-based forest fire detection method, GXLD, is proposed. GXLD uses the dark channel prior to defog the image to obtain a fog-free image. After the lightweight improvement of YOLOX-L by GhostNet, depth separable convolution, and SENet, we obtain the YOLOX-L-Light and use it to detect the forest fire in the fog-free image. To evaluate the performance of YOLOX-L-Light and GXLD, mean average precision (mAP) was used to evaluate the detection accuracy, and network parameters were used to evaluate the lightweight effect. Experiments on our forest fire dataset show that the number of the parameters of YOLOX-L-Light decreased by 92.6%, and the mAP increased by 1.96%. The mAP of GXLD is 87.47%, which is 2.46% higher than that of YOLOX-L; and the average fps of GXLD is 26.33 when the input image size is 1280 × 720. Even in a foggy environment, the GXLD can detect a forest fire in real time with a high accuracy, target confidence, and target integrity. This research proposes a lightweight forest fire detection method (GXLD) with fog removal. Therefore, GXLD can detect a forest fire with a high accuracy in real time. The proposed GXLD has the advantages of defogging, a high target confidence, and a high target integrity, which makes it more suitable for the development of a modern forest fire video detection system.

## 1. Introduction

Forest fire, as one of the most frequent and serious natural disasters, not only destroys the forest, but also causes extensive damage to the ecosystem [1]. Forest fire occurs frequently in China. According to the statistics of the Fire Rescue Bureau of the Ministry of Emergency Management of China, 616 forest fires in China destroyed approximately 4292 hectares of forest just in 2021 [2]. If a forest fire is not detected in time, it can easily cause an uncontrollable disaster, resulting in more casualties and economic losses [3]. Therefore, accurate, efficient, and timely forest fire detection is imperative to prevent the forest fire.

At present, several forest fire detection methods have been implemented, such as manual patrol [4], the satellite remote sensing-based method [5], and the video monitoring-based method [6]. Among them, manual patrol requires the forest ranger to continuously patrol the forest and report the fire in time [7]. However, the patrol area is limited, and it is difficult to achieve all-weather monitoring [8]. Optical satellite remote sensing can detect a forest fire in a wide spatial range; however, it is difficult to monitor the forest fire with a high spatial resolution in real time due to the conflict between the spatial and temporal resolution of the satellite remote sensing systems [9]. 

Forest fire detection with video is a technology based on computer vision [6]; compared with manual patrolling, video monitoring allows the real-time detection of forest fires in large areas and all-weather conditions. Compared with satellite monitoring, video monitoring can accurately detect forest fires at their initial stage. Therefore, a video surveillance system is more effective at detecting forest fires [10]. 

There are two categories of video-based forest fire detection methods: traditional algorithms and deep learning-based methods. The traditional forest fire detection algorithm is based on artificially designed smoke and flame features to detect forest fires, which can effectively detect an early stage of fires. However, this method has a too complex feature design and relies on prior knowledge, resulting in poor accuracy and real-time performance. With the rapid development of deep learning, various convolutional neural networks (CNN) models, such as you only look once (YOLO) and the single shot multi-box detector (SSD), have been introduced to develop forest fire detection algorithms [11,12,13,14,15]. Compared with the traditional forest fire detection algorithms, CNN-based forest fire detection methods can directly output the final detection results according to the features that learned by the network. In addition, deep learning-based methods can accurately detect forest fires in their early stage. 

However, the CNN-based forest fire detection algorithms also have obvious limitations. It must be noted that training CNN-based target detection algorithms requires a substantial amount of training data [16]. In addition, the current open-source forest fire dataset was built with a low image resolution, short shooting distance, and large fire intensity. The models trained with the above datasets can hardly meet the requirements for high image resolution, long monitoring distance, and early stage fire detection in forest fire prevention and control [17]. In addition, the CNN-based model has a large number of parameters, which require higher computing power to ensure real-time detection [18]. 

The detection of forest fire is easily disturbed by fog. The study area (see Section 2.1) in this paper is characterized by complex terrain and fog. In a foggy environment, the reflected light of the shooting target will be absorbed, refracted, and scattered by the suspended particles in the air, resulting in the attenuation of natural light. This may lead to the overall whiteness, contrast reduction, and the color deviation of the captured image [19]. Moreover, the targets may be covered by fog [20], which will result in poor detection performance. Smoke has similar physical characteristics to fog (both have a white color and foggy shape), which also results in reducing the accuracy of the forest fire detection. What is more, there are many people living in mountain forests of the study area; we can’t ignore the possibility of the main fire sources being caused by their productive and living activities [21].

Taking the above problems into account, this paper presents a lightweight forest fire detection method based on the YOLOX-L model and defogging method. First, GhostNet is introduced to replace the Backbone network of YOLOX-L, partly reducing the overall network parameters. Then, we integrate the efficient squeeze-and-excitation (SE) attention mechanism at the Backbone output to enhance the ability of network in feature extraction. Finally, some ordinary convolutions in neck and prediction are replaced by deeply separable convolutions, which greatly reduce the parameters of the network and improve the network detection speed. These improvements enhance the ability of network in feature extraction. In addition, dark channel prior (DCP), which can reduce fog interference and improve method performance is introduced to remove fog from the video and images. To evaluate the performance of the proposed method for forest fire detection in real time, the proposed method and other methods are developed and trained with a high-quality forest fire dataset that is built based on the open-source online forest fire data; the data collected during the planned burning period in Mianning County and Xide County, Liangshan Prefecture, Sichuan Province; and the video monitoring data provided by the local forestry and grass bureau. The solution of these key problems will effectively improve the forest fire detection performance of the forest fire video detection system and reduce the system construction cost.

## 2. Materials and Methods

### 2.1. Study Area

**Figure 1:**
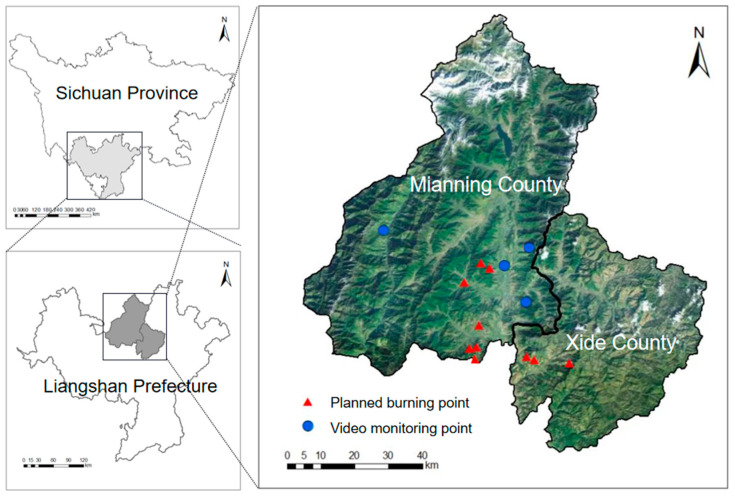
Location of study area and data collection points in Mianning County and Xide County. The background color map is the study area (Mianning County and Xide County) and its location.

The study areas in this paper are Mianning County and Xide County, Liangshan Prefecture, Sichuan Province, China. Their geographical locations are shown in Figure 1. Mianning County and Xide County are located at the mountainous area in the southwest of Sichuan Basin, belonging to the subtropical monsoon climate zone. The average altitude of the whole region is over 1500 m. The solar radiation is high during the day and the temperature difference between day and night here is huge. Every year from January to June, with hot dry weather and little precipitation for a long time, forest fires are easier to occur. Mianning County and Xide County plan to start the planned burning in January 2022, allowing us to collect a lot of real and effective forest fire data in a month.

### 2.2. Forest Fire Dataset

**Figure 2:**
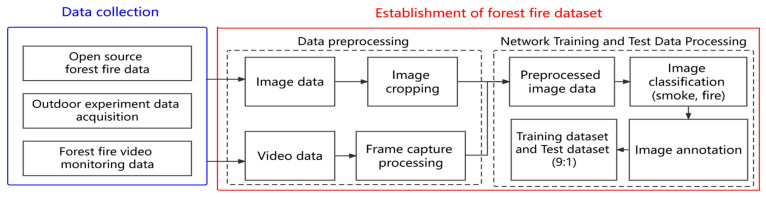
The data processing flow for forest fire dataset.

The specific process of data processing for the forest fire dataset is shown in Figure 2. Data collection in this study mainly includes open-source forest fire data, field experiment data, and forest fire video monitoring data provided by the local forestry and grass bureau. Establishment of the forest fire dataset mainly includes data preprocessing, network training, and test data processing; we will introduce the data processing procedure in detail.

#### 2.2.1. Data Collection

In order to obtain more data in the early stage of forest fires, the data were collected from the planned burning areas of Mianning County and Xide County from January 4 to January 6, 2022. To ensure data diversity, multi-angle and distance data were captured with a digital single-lens reflex (DSLR) camera and an unmanned aerial vehicle (UAV); the shooting distance of camera is set between 2–5 km and the UAV flight altitude is kept between 50–150 m. The specific information of the capture device is shown in Table 1.

We captured 103 videos and 115 images, with a total size of 10.0 GB. The specific information of the 11 captured areas is shown in Table 2. During the whole collection process, the weather was sunny on January 4 and 5, but cloudy and foggy on January 6. The hand-held GPS equipment was used to record the longitude and latitude of each capture location.

In addition, 205 forest fire monitoring videos with a total size of 94.7 GB were acquired from the local forestry and grass bureau during December 2021 to January 2022. The specific information of the four areas captured by the video monitoring system is shown in Table 3.

The open-source forest fire data of the national laboratory of fire science, University of Science and Technology of China [22] and Bilkent EE Signal Processing group [23] were screened to obtain 1147 images of high image resolution, long monitoring distance, and early stage fire detection in forest fire prevention and control that are suitable for this experiment.

#### 2.2.2. Establishment of Forest Fire Dataset

**Figure 3:**
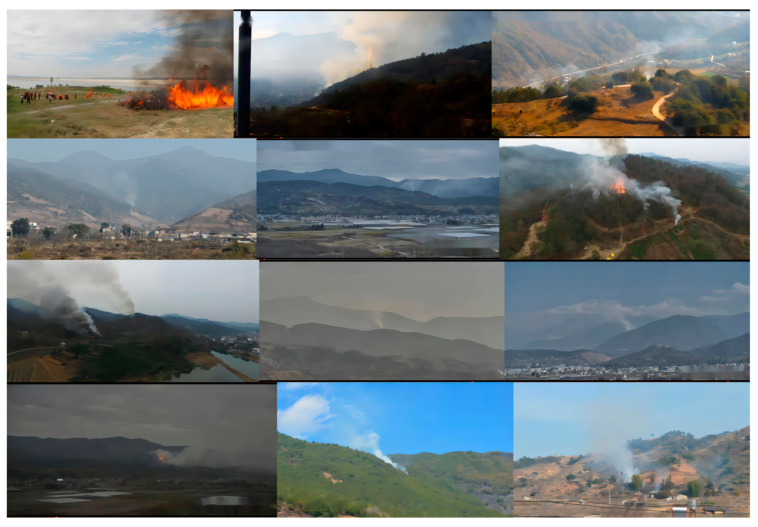
Example of part of the used data.

In the phase of data preprocessing, the image data is cropped to remove the watermark and fuzzy areas in the image. We take a screenshot of the video every 3 min to obtain more image data with different smoke shapes. Because the image obtained from the screenshot is a real forest fire image, in order to make full use of such data, the image obtained from the screenshot will not be cropped. Part of the used data are shown in Figure 3. The specific information of the dataset is shown in Table 4.

In the phase of network training and test data processing, the open-source tool LabeImg is used to label images, and divide the dataset into a training set and test set with a 9:1 ratio, which is used for the network training and test. To ensure the validity of the test set, we designed forest fire images with different scenes as the data in the test set.

### 2.3. The Proposed Forest Fire Detection Method

**Figure 4:**
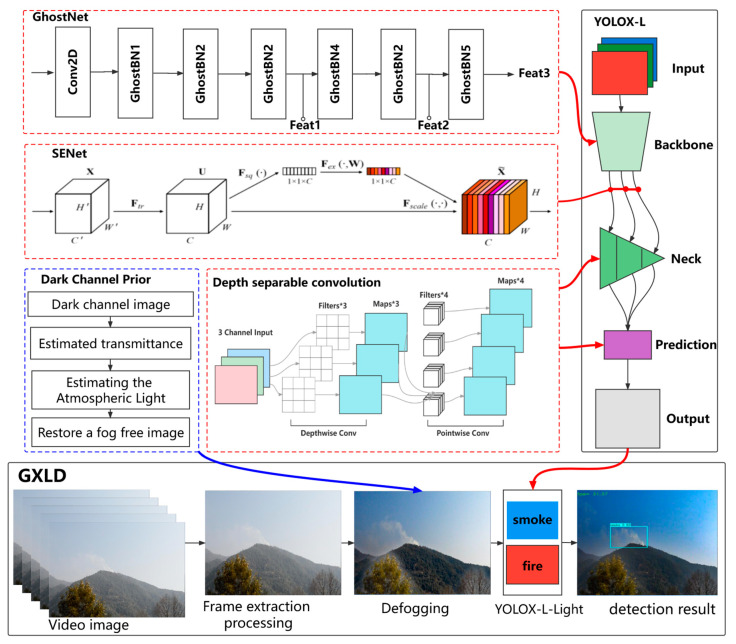
Overall design process of GXLD, including GhostNet network structure, SENet structure, DCP defogging process, and YOLOX-L network structure.

The overall design process of GXLD (GhostNet-YOLOX-L-Light-Defog) is shown in Figure 4. The core parts of GXLD are YOLOX-L-Light and the dark channel defogging method. YOLOX-L-Light is the result of light-weighted YOLOX-L, which included the introduction of GhostNet to replace the Backbone network, the improvement of some ordinary convolutions in neck and prediction to deeply separable convolutions, and the integration of the SE attention mechanism at the Backbone output. The dark channel defogging method, which is based on the dark channel prior, mainly obtains the fog-free image by calculating the dark channel image, estimating the transmittance, and calculating the atmospheric light value. These two core parts are described in detail below.

#### 2.3.1. YOLOX-L-Light

YOLOX network is a new target detection framework proposed by Broadview in 2021 [24], which is mainly based on the improvement of YOLOv3 network. The network improvement mainly includes backbone network structure, classification and regression decoupling head, anchor free frame mechanism and dynamic matching positive samples. YOLOX network is composed four modules: Input, Backbone, Neck and Prediction. Two powerful data enhancement technologies Mixup [25] and Mosaic [26] are mainly used at the input. Mosaic can effectively improve the detection effect of small targets. Mixup is an additional enhancement strategy based on Mosaic. The Backbone of YOLOX network is consistent with that of the original YOLOv3 [27] network, and the Darknet53 network is adopted. The Neck part also adopts the Feature Pyramid Networks (FPN) structure for integration. Prediction consists of decoupling head, anchor free detector, tag allocation strategy and loss calculation. YOLOX can be divided into standard network structure and lightweight network structure by adjusting the width and height of the network. In this paper, YOLOX-L network with the best performance in the standard network structure is selected, and makes lightweight improvement is get YOLOX-L-Light.

**Figure 5:**
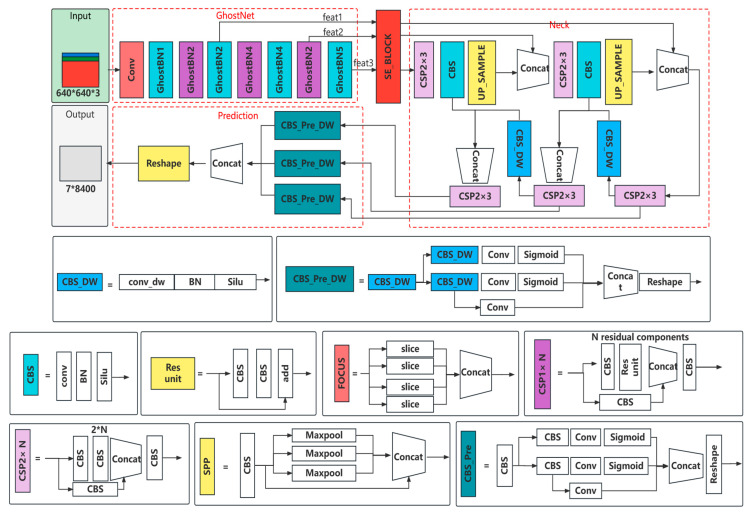
YOLOX-L-Light network structure.

The lightened YOLOX-L model (YOLOX-L-Light) is shown in Figure 5. Firstly, we replace the Backbone of YOLOX-L network with GhostNet. GhostNet [28] network has advantages of maintaining the recognition performance of similarity and reducing convolution operation. The GhostNet can surpass MobileNet [29] and SSD [30] in accuracy and efficiency with relative low network parameters. We use GhostNet as the feature extraction network of YOLOX-L. As shown in Figure 5, the Conv in the GhostNet represents two-dimensional convolution of the input feature map, Ghost BN represents Ghost Bottle Neck, which is the basic unit of GhostNet. Feat1, Feat2, and Feat3 represent feature map with three scales respectively, which include 80 × 80 × 40, 40 × 40 × 112 and 20 × 20 × 160. The output is input into Neck for feature extraction in the next step.

To further reduce the parameters of YOLOX-L, we replace normal convolution with depth separable convolution. Depth separable convolution [29] is different from ordinary convolution in that it consists of depth convolution and pointwise convolution. Previous studies have proved that the replacement of ordinary 3 × 3 convolution in the CNN with depth separable convolution can effectively reduce the amount of network parameters [29]. This paper refers to the position of depth separable convolution in the YOLOX-nano model to replace some ordinary convolutions in neck and prediction in YOLOX-L. The specific location of the CBS_DW module is shown in Figure 5.

The attention mechanism is a structure to improve the network’s attention to the space and channel information of features. The accession of the attention mechanism can strengthen the network structure’s ability to extract key features in innumerable feature information. Thus, the network’s performance is greatly enhanced [31]. At present, the mainstream attention mechanisms can be divided into the following three types: channel attention, spatial attention [32], and self-attention [33]. SENet is a typical channel attention mechanism [34]; it can strengthen the relationship between channels concerned by the network. So, the weight of the feature information concerned on the feature layers of different channels is various. As a plug and play module, the attention mechanism can, in theory, be placed behind any feature layer. The SENet is introduced in this study to extract the important features in the output of Backbone.

#### 2.3.2. Defogging Using Dark Channel Prior Theory

Dark channel prior theory was first proposed by He et al. [35]. They obtained a prior rule through experimental results on a large number of fog-free images. This rule states that in most clear fog-free color images, after removing the sky part and some areas with high brightness, there must be a color channel in the local non-haze area that contains a large number of pixels (called dark pixels) with an intensity of about 0. This channel is named as the dark channel, which is defined as Equations (1) and (2):

(1) $$J^{\mathrm{dark}}\left(x\right)=\underset{y\in \Omega \left(x\right)}{\min}\left(\underset{c}{\min}J^{c}\left(y\right)\right),c\in \left(R,G,B\right)$$

(2) $$J^{\mathrm{dark}}\left(x\right)\to 0$$

where $J^{dark}$ is the dark channel image. Ω(x) represents the area around the pixel point *x*; $J^{c}$ is a channel in the fog-free image. c is the visible light image, including red, green, and blue color components.

The image taken by the camera consists of the following two parts. The first part is the reflected light of the shooting target; however, it may be attenuated due to the scattering and absorption of atmospheric light. The other part is atmospheric light after being scattered. The formula of the atmospheric scattering model can be expressed as Equation (3):

(3) I(t)=J(x)t(x)+A(1−t(x))

where *I(t)* is the foggy image, *J(x)* is the fogless image, *A* is the atmospheric light value, and *t(x)* is transmissivity.

According to the prior rule of the dark channel image and the combination with the atmospheric scattering model, fog, which is *J(x)* in Equation (3), can be removed. Assuming that the transmittance of the same area remains unchanged and the atmospheric light value A is known, Equation (3) can be divided by the atmospheric light value to obtain Equation (4):

(4) $$\frac{I^{c}\left(x\right)}{A^{c}}=t\left(x\right)\frac{J^{c}\left(x\right)}{A^{c}}+1-t\left(x\right)$$

Both sides of Equation (4) are minimized to make them approach to the dark channel (Equation (5)):

(5) $$\underset{y\in \Omega \left(x\right)}{\min}\left(\underset{c}{\min}\frac{I^{c}\left(y\right)}{A^{c}}\right)=\tilde{t}\left(x\right)$$

where $\tilde{t}\left(x\right)$ is a constant in the area around pixel *x*; thus, it is not minimized. $A^{c}$ is the atmospheric light value of color channel *c*, and *J* is the fog-free image to be obtained. Combining Equations (1) and (2), we can deduce Equation (6):

(6) $$\tilde{t}\left(x\right)=1-\underset{y\in \Omega \left(x\right)}{\min}\left(\underset{c}{\min}\frac{I^{c}}{A^{c}}\right)$$

In order to make the defogged image more natural, it is necessary to increase the depth of field information in the image. Therefore, a constant coefficient ω is introduced into Equation (6); after that, a rough transmittance can be obtained by means of Equation (7):

(7) $$\tilde{t}\left(x\right)=1-\omega \underset{y\in \Omega \left(x\right)}{\min}\left(\underset{c}{\min}\frac{I^{c}}{A^{c}}\right)$$

where ω is usually set as 0.95.

A common method for estimating the atmospheric light value $A^{c}$ is to directly take the maximum value of pixel intensity from an image. This method is not only simple, but also effective. However, the outdoor image may contain a large proportion of sky areas or gray-white objects, which will cause a dramatic interference to the estimation of pixel intensity, and result in a large deviation between the estimated atmospheric light value and the real scene. The dark channel defogging method first extracts the pixel values of the first 10% with the lowest intensity from the previously obtained transmittance image. These pixels have the maximum fog concentration at the same time, and their gray value can be approximately equivalent to the atmospheric illumination value.

The transmissivity t(x) and atmospheric light value *A* are obtained from the previous steps. The fogless image can be recovered by substituting the two values into Equation (8):

(8) $$J\left(x\right)=\frac{I\left(x\right)-A}{\max(t\left(x\right),t_{0})}+A$$

where $t_{0}$ is the minimum of transmissivity. In order to prevent the overall whitening of the image due to the small value of t(x), it is generally set as 0.

**Figure 6:**
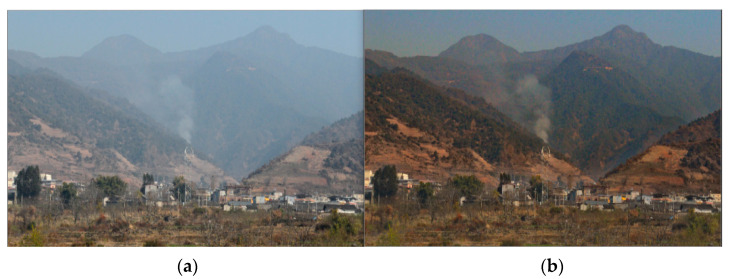
Defogging effect: (**a**) is the original image and (**b**) is the image after defogging.

The defogging result of the method is shown in Figure 6. The dark channel defogging method can better defog the image and retain the characteristics of thick smoke, which will provide less fog interference images for subsequent forest fire detection.

### 2.4. Experimental Setting and Evaluation Index

In this study, all the experiments were performed using an Intel Core i7-8700 with 16 GB RAM on a platform of a Windows 10, 64 bit operating system; and an NVIDIA Geforce RTX3060 graphic card having 12 GB of VRAM. The proposed model is implemented with the PyTorch 1.2.0 deep learning framework. The device configuration we used in this experiment is as Table 5.

During the experiment, we use the same hyperparameter to train YOLOX-L-Light, YOLOX-L, YOLOX-Tiny, YOLOv4, and YOLOv4-Tiny. The specific values of the hyperparameter are listed in Table 6. In addition, we also add YOLOv4-Light proposed by FAN [36], and train it with the same hyperparameter.

To evaluate the performance of network models and GXLD, mean average precision (mAP) in Pascal VOC was used to evaluate the detection accuracy. mAP is the average value obtained after calculating the average precision (AP) for each category. AP is a general evaluation index in target detection, which can assess the accuracy of classification and positioning. Classification is to judge whether the prediction is smoke and flame, and positioning is to judge whether the intersection of the union (IoU) between the network prediction box and the manual label box meets the requirements. The AP value is equivalent to the area under the recall and precision curves, where the precision and recall are defined as Equations (9) and (10):

(9) $$\mathrm{Pre}cision=\frac{T_{P}}{N_{d}}$$

(10) $$\mathrm{Recall}=\frac{T_{P}}{N_{g}}$$

where $T_{P}$ is the number of real classes in the detection results, and $N_{d}$ is the number of detection boxes after non maximum suppression; $N_{g}$ is the number of dimension boxes.

## 3. Results and Discussion

### 3.1. The Experiment Results of the YOLOX-L-Light

Firstly, the quantity of model parameters is evaluated, which is calculated by using the summary module under the Python deep learning framework. The results are shown in Table 7. The results indicate that the parameters of the YOLOX-L-Light model are not only smaller than YOLOX-L, YOLOv4, and YOLOv4-Light, but also smaller than those of the official lightweight YOLOX-Tiny and YOLOv4-Tiny. It shows that the proposed lightweight strategies can greatly reduce the number of network parameters.

In order to compare the average precision (AP) and the mean average precision (mAP) of each model, we tested all the trained models on the same dataset, and the statistical results of detection accuracy are shown in Table 7. According to the results, the mAP of all models is more than 0.85, indicating that the models work well on forest fire detection. Among them, YOLOX-L-Light has the highest mAP (86.81%), which is 1.8% higher than YOLOX-L and 0.78% higher than YOLOx4-Light. In addition, the AP of each category of YOLOX-L-Light is higher than that of other models. The accuracy of its flame category is 84%, and the accuracy of its smoke category is 89.62%. The results indicate that the improved lightweight network YOLOX-L-Light can effectively increase the accuracy of forest fire detection with fewer parameters.

Ablation experiments were conducted for the improved structure to demonstrate the effectiveness of each of the proposed improvements to the YOLOX-L network. The experimental results are shown in Table 8, where GhostNet-YOLOX-L-dsc is the network obtained by introducing the GhostNet network and deeply separable convolution into YOLOX-L. GhostNet-YOLOX-L-SE is the network obtained by introducing the GhostNet network into YOLOX-L and integrating the SE attention mechanism. YOLOX-L-dsc-SE is the network obtained by introducing the deeply separable convolution into YOLOX-L and integrating the SE attention mechanism.

The results of ablation experiments indicate that the introduction of GhsotNet can effectively improve the accuracy of the network and reduce most of the network parameters. The introduction of deep separable convolution can effectively reduce some network parameters without reducing the accuracy of the network. The introduction of the SE attention mechanism can effectively improve the network accuracy.

### 3.2. The Experment Results of the GXLD

**Figure 7:**
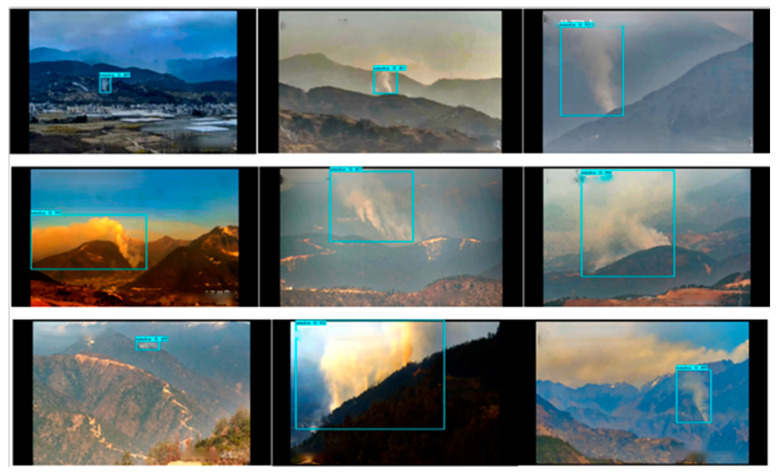
Detection results of fog environment of the GXLD.

We tested GXLD on the test dataset and obtained the statistical results of detection accuracy as shown in Table 9. The mAP of GXLD is 87.47%, which is 2.46% higher than the original YOLOX-L and 0.66% higher than YOLOX-L-Light. The specific detection results are shown in Figure 7.

In order to verify the real-time performance of GXLD, we manually selected 12 video monitoring data and 12 camera shooting data for the FPS test of GXLD, for which, including 11 videos of the fog environment, the average duration of each video is 3 min, and the average original FPS of each video is 29.33. We adjusted the input image sizes to 1280 × 720 and 720 × 480, respectively, using the resize operation in OpenCV; the frame number of GXLD frame extraction processing is adjusted to 8, which means detecting one frame every eight frames. As shown in Table 10, when the input image size is 1280 × 720, the maximum FPS of GXLD is 30.51, the minimum FPS is 25.14, and the average FPS is 26.33. When the input image size is 720 × 480, the maximum FPS of GXLD is 68.12, the minimum FPS is 50.51, and the evaluation FPS is 56.41. This shows that GXLD can realize real-time detection when the input images are 1280 × 720 and 720 × 480.

**Figure 8:**
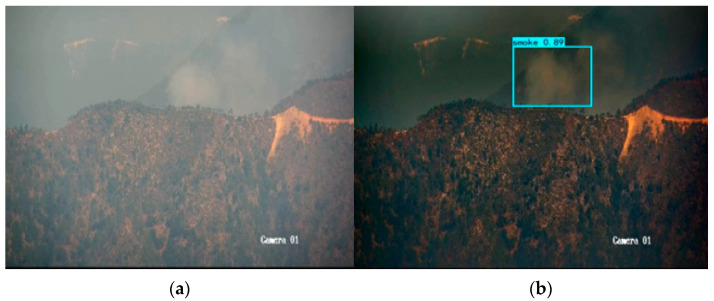
Comparison of forest fire detection result in foggy environment by (**a**) YOLOX-L-Light and (**b**) GLXD.

**Figure 9:**
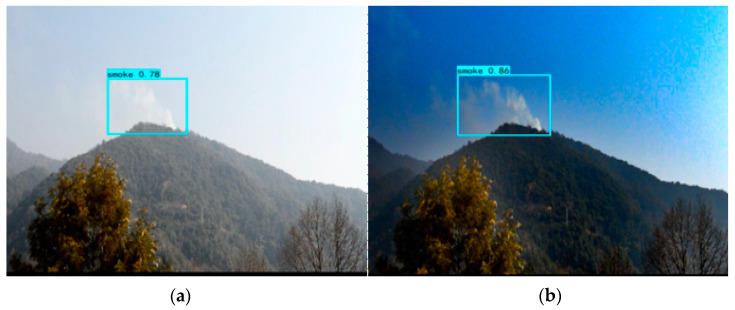
Comparison of target confidence of forest fire in foggy environment by (**a**) YOLOX-L-Light and (**b**) GLXD.

According to the specific data in Table 9 and Table 10, GXLD has excellent forest fire detection effect and real-time detection capability. In addition, GXLD also has certain advantages in target confidence and target integrity. The left figure in Figure 8 and Figure 9 shows the detection results of YOLOX-L-Light, and the right figure shows the detection results of GXLD.

It can be seen from Figure 8 that, at the same time, GXLD can effectively detect smoke, while YOLOX-L-Light cannot. In Figure 9, although both models have detected smoke, GXLD’s target confidence is 0.86, while YOLOX-L-Light’s target confidence is 0.78. In terms of target integrity, GXLD can display more complete smoke and frame it.

## 4. Conclusions

This research proposes a lightweight forest fire detection method (GXLD) with fog removal. GXLD can achieve real-time and high accuracy forest fire detection. It has the advantages of defogging, a high target confidence, and a high target integrity, which are more suitable for the development of a modern forest fire video detection system.

First, a high-quality forest fire dataset is built with open-source datasets, an outdoor experiment dataset, and a video monitoring data system. Then, a lightweight method YOLOX-L-Light model is proposed by improving YOLOX-L. With the same hyperparameter, we trained and tested YOLOX-L-Light, YOLOX-L, YOLOV4, YOLOV4 Tiny, and YOLOV4-Light. Experiment results show that the proposed YOLOX-L-Light outperforms other models in terms of both precision (mAP = 86.13%) and parameter quantity (about 4 MB). The ablation experiment proved that the proposed lightweight strategies can significantly reduce the number of network parameters and enhance the network feature extraction ability.

In addition, this study combines YOLOX-L-Light with the dark channel defogging method to obtain GXLD and evaluate its performance. The results show that the mAP of GXLD on the test dataset is 87.47%. The average fps is 26.33 when the input image size is 1280 × 720. GXLD also has excellent performance in target confidence and target integrity.

In the experiment, we also found there are still some limitations in GXLD. The detection performance of GXLD is poor in a very serious dense foggy scene. The future research will make more in-depth lightweight improvement on YOLOX-L-Light and needs to conduct more in-depth research on the defogging method; thus, to achieve a forest fire detection method with better performance and serve the forest fire prevention and control.

## Figures and Tables

**Table 1:** Capture device information.

Capture Device	Maximum Resolution	Image Sensor
NIKON D3200	6016 × 4000	1/2.3 inch CMOS sensor
DaJiang Mavic Air 2	8000 × 6000	1/2 inch CMOS sensor

**Table 2:** Information of planned burning area.

Planned Burning Area	Acquisition Time	Capture Latitude and Longitude
Zhuang Village, Hebian Town, Mianning County	4 January 2022	102°4′26.159″ E, 28°20′6.144″ N
Dashuigou Village, Zeyuan Town, Mianning County	5 January 2022	102°7′2.388″ E, 28°23′1.194″ N
Guodi Village, Hebian Town, Mianning County	5 January 2022	102°8′30.372″ E, 28°22′18.386″ N
Er Village, Zeyuan Town, Mianning County	5 January 2022	102°6′44.057″ E, 28°13′20.176″ N
Jiaoding Village, Manshuiwan Town, Mianning County	5 January 2022	102°6′18.652″ E, 28°9′51.512″ N
Jiaoding Village, Manshuiwan Town, Mianning County	5 January 2022	102°5′15.526″ E, 28°9′36.608″ N
Jiaoding Village, Manshuiwan Town, Mianning County	5 January 2022	102°6′18.662″ E, 28°9′49.705″ N
Guangming Village, Hongmo Town, Xide County	6 January 2022	102°21′7.956″ E, 28°7′17.033″ N
Luji Village, Hongmo Town, Xide County	6 January 2022	102°15′33.192″ E, 28°7′51.722″ N
Luji Village, Hongmo Town, Xide County	6 January 2022	102°14′19.725″ E, 28°8′19.167″ N
Madebao Village, Manshuiwan Town, Mianning County	6 January 2022	102°6′5.411″ E, 28°7′54.106″ N

**Table 3:** Video-monitoring area information.

Video-Monitoring Point	Acquisition Time	Longitude and Latitude
Mountainous area of Hongmo Town, Mianning County	17 December–20 December 2021	102°10′50.293″ E, 28°22′45.588″ N
Houshan, Mianning County	29 December 2021	102°14′45.215″ E, 28°25′35.195″ N
Tiekuangshan, Lugu Town, Mianning County	24 December–30 December 2021	102°14′14.107″ E, 28°16′55.394″ N
Luning Mountain Area, Jinping Town, Mianning County	04 January 2022	101°51′34.042″ E, 28°28′17.440″ N

**Table 4:** Forest fire dataset.

Forest Fire Dataset	Quantity of Fire Data	Quantity of Smoke Data	Quantity of Data Containing both Smoke and Fire	Total Quantity
Forest Fire Images	512	533	1113	2158

**Table 5:** Experimental device configuration.

Device	Configuration
CPU	Intel(R) Core(TM) i7-8700 CPU @ 3.20 GHz 3.19 GHz
RAM	16 GB
GPU	NVIDIA Geforce RTX3060 12 G
IDE	VScode
Others	Pytorch, CUDA11.0, CuDNN8.0, Anaconda, opencv4.40

**Table 6:** Hyperparameter.

Hyperparameter	Value
Training set	1968
Test set	223
Iterations	19,200
Epochs	200
Maximum learning rate	0.261
Minimum learning rate	0.00261
Batch size	16

**Table 7:** Comparison of the performance of different CNN models.

Network	Parameter	mAP	AP_Fire	AP_Smoke
YOLOX-L-Light	3,994,609	86.81%	84%	89.62%
YOLOX-Tiny	5,033,157	85.21%	80.64%	89.59%
YOLOv4-Tiny	5,876,426	85.20%	81.69%	88.71%
YOLOv4-Light	12,615,535	86.03%	87.26%	84.81%
YOLOX-L	54,148,757	85.01%	82.09%	87.93%
YOLOv4	63,943,071	85.91%	83.86%	87.78%

**Table 8:** The results of each lightweight improvement method.

Network	Parameter	mAP
YOLOX-L-Light	3,994,609	86.81%
GhostNet-YOLOX-L-dsc	3,989,681	86.13%
GhostNet-YOLOX-L-SE	10,363,753	86.77%
YOLOX-L-dsc-SE	45,450,133	85.59%
YOLOX-L	54,148,757	85.01%

**Table 9:** The performance of GXLD for forest fire detection.

Method	mAP	AP_Fire	AP_Smoke
GXLD	87.47%	85%	90%

**Table 10:** FPS of GXLD of different input image sizes.

Input Image Sizes	Quantity of Video	Max_FPS	Min_FPS	Average_FPS
1280 × 720	24	30.51	25.14	26.33
720 × 480	24	68.12	50.51	56.41

## Data Availability

Not applicable.
